# Washington University of Medicine

**Published:** 1869-04

**Authors:** 


### ARTICLE IX.

### *Washington University of Medicine*, Baltimore Md.

The Second Annual Commencement of this College was held at Holliday Street Theatre, February 22nd. The degeee of "Doctor of Medicine" was conferred upon eighty-two gentlemen, representing fourteen States, by Dr. Edward J. Chaisty; Professor Thos. E. Bond, the President of the Faculty, being unavoidably absent. The Valedictory Address was delivered by Professor Charles W. Chancellor, Dr. J. Carter Cook, of Georgia. responded in behalf of the students. Professor Clagett, acting dean, read the report of Professor Noel, of the Baltimore College of Dental Surgery, and Drs. Littell and Post, the Committee selected to examine the theses of the graduating class, and to award the prize of $100. offered by the Faculty. This report stated that the papers of Alonzo Sims of Mississippi, and R. S. Hart, of Alabama were the best, and of such equal merit as to necessitate a division of the prize. Dr. E. J. Henkle President of the Board of Visitors, then presented the prizes. An address was also delivered by Maj. A. B. Magruder, late of Virginia, now of Baltimore.

